# *Optimeet*: A computational tool to enhance participant attendance in group research

**DOI:** 10.3758/s13428-025-02745-9

**Published:** 2025-09-17

**Authors:** Lukas W. Mayer, Desislava Bocheva, Joanne Hinds, Olivia Brown, Lukasz Piwek, David A. Ellis

**Affiliations:** 1 Department of Cognitive Sciences, UCI School of Social Sciences, University of California Irvine, Irvine, CA 92617 USA; 2 Decisions and Operations Division, School of Management, University of Bath, Claverton Down, Bath, BA2 7AY UK

**Keywords:** Participant recruitment and scheduling, Group research, Statistical power, Simulation

## Abstract

Across disciplines, research often relies on groups of people to participate in experiments or attend events at the same time. Typically, researchers try to maximize attendance by manually identifying a set of times that suit the diaries of many individuals. However, this is inefficient, is prone to error, and can lead to a final sample that is not large enough to provide meaningful inferences. While current scheduling tools are useful for individual-based research, enabling participants to select times convenient to them within a researcher's preset parameters, they are less useful in research that requires specific or flexible group sizes. In response, we present *Optimeet*, a web application that allows researchers to upload participants’ availability data and generate an optimal allocation schedule for multiple groups. We describe the function of the underlying applet, which identifies a schedule to maximize attendance by treating it as a computational problem involving combinatorial optimization (Experiment 1). Our solution relies on an empirical comparison of parameter-free heuristics to make allocation decisions that make the best use of participants’ availabilities and the derivation of appropriate performance metrics. Of the algorithms evaluated, one consistently outperformed comparable versions of existing tools, which we verified in a further exercise (Experiment 2) involving a large human sample (*N* = 5,289). We consider the methodological utility and practical value of these developments, and include detailed documentation, code, and a video tutorial so that researchers can rapidly employ *Optimeet* to support group research.

**Supplementary Information:**

The online version contains supplementary material available at 10.3758/s13428-025-02745-9.

## Introduction

Organizing individuals into groups and coordinating their participation often constitutes a substantial component of studies within the psychological, behavioral, and social sciences. For example, empirical investigations of groups and teams can be found in fields as diverse as behavioral game theory (Hawkins, 2015), evidence-based social policy (Balafoutas & Sutter, 2012; Hauser et al., 2014; Janssen et al., 2010; Nishi et al., 2015), cumulative cultural evolution (Derex et al., 2013), psychotherapy (Joyce et al., 2010; Rebok et al., 2014), education (Osborne & Collins, 2001; Yeager et al., 2019), market research, and organizational psychology (Spoon et al., 2021). Other long-standing paradigms involving clinical trials, focus groups (Krueger, 2014), and experiments (Van Vugt & Hart, 2004; Wölfer et al., 2015) regularly require the allocation of participants into groups and the scheduling of appointments. Irrespective of the area, researchers can encounter difficulties in participant recruitment and securing sufficient sample sizes (Schmutz et al., 2019). Typically, these challenges arise from participants having other commitments, having conflicting or inflexible schedules in relation to a study's requirements, or simply deciding to not attend (Khatamian Far, 2018; Powell & Single, 1996).

At the same time, an increasing volume of research is hosted in online environments. Participant recruitment platforms such as Prolific, Amazon Mechanical Turk (MTurk), and UserTesting, as well as a variety of other social media and citizen science platforms (e.g., Zooniverse), have made the recruitment of large samples for both online and offline group research more accessible (Gallo & Yan, 2015; Sauter et al., 2020). These platforms offer other notable advantages to group researchers, but they are most effective for simple study designs in which participants are immediately assigned to a group for a short, self-contained task. For instance, online platforms have been used to study social identity processes by rapidly assigning individuals into “chat rooms” with other participants (O’Reilly et al., 2022). Unfortunately, these platforms are less able to accommodate more complex designs that involve allocating individuals to groups and scheduling their participation in sessions that involve interaction with others, whether online, in person, or a hybrid of the two. The platforms are only designed to handle single-slot appointments or single-slot group appointments (Chandler et al., 2019; Peer et al., 2022).

Researchers conducting appointment-based group research in person or online continue to face challenges when allocating individuals to slots that also accommodate the availability of other participants. A prevailing issue becomes securing enough groups to meet a desired or predetermined sample size and achieve adequate statistical power,^1^ because the primary unit of analysis in such studies is the *group*, rather than the *individual* (Laughlin, 2011). Although the issue of statistical power should be addressed ideally through a priori design decisions, inefficient scheduling may result in having to recruit more participants than necessary to ensure the required number of groups. For example, having, for this reason, to recruit 500 participants rather than the 300 needed to achieve a final sample of 60 five-member groups slows research progress severely and places extra burdens on limited research resources. Given ongoing budget constraints, this issue may limit researchers in the studies they can realistically conduct, making it crucial that the allocation of individuals to time slots is optimized and group participation is scheduled more effectively.

In this paper, we address these challenges through *Optimeet*, a web application designed to improve appointment scheduling for group-based studies. We provide an overview of *Optimeet*’s features and validate its effectiveness through simulations and human trials. In the following section, we discuss the limitations of existing methods and explain how *Optimeet* offers a more efficient solution for group-based scheduling.

### Existing methods that aim to enhance attendance

Many researchers start by devising their own strategies for manually allocating individuals to groups, such as asking participants to arrange an appointment between themselves or employing spreadsheets or more task-specific commercial tools (Bond et al., 2018; see Table 1). Tools such as *Sona Systems*, *Doodle*, and *Calendly* are useful for individual-based research, allowing participants to select convenient times within researchers’ preset parameters (Alrawi et al., 2016). However, these solutions become inefficient in group-research contexts because they require participants to self-select single appointments and to indicate their availability across other time slots too.

**Table 1:**
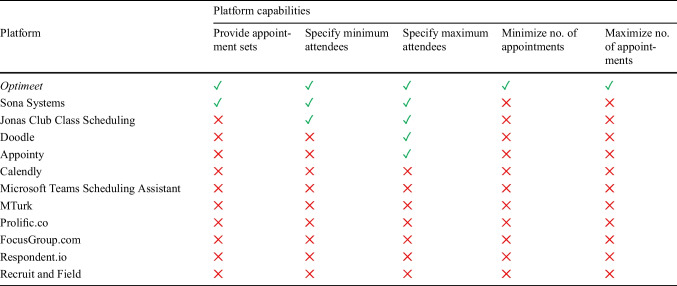
Overview of features provided by *Optimeet* (described here) and other appointment-scheduling platforms

*Note.* This table compares the key capabilities of each platform with *Optimeet,* which uses our best-performing algorithm (Minimax). It does not provide an exhaustive review of all features that may be better suited to certain contexts, such as scheduling individual meetings or crowdsourcing studies

This inefficiency can be exacerbated in group-research settings because there is often a need to form groups of predefined sizes and configurations, which may entail (1) a specific size, (2) a flexible size (e.g., ranging from three to five members), or (3) an open-ended size (e.g., three or more members). For example, if researchers needed to recruit focus groups of four to six members each, they might aim to maximize participant attendance by minimizing the number of appointments offered or, alternatively, might maximize the number of appointments offered in an attempt to be more flexible and thereby maximize attendance. Participants may find that their desired sessions are already at capacity, or they might inadvertently select an appointment that does not prove popular enough to be feasible (e.g., only two participants sign up). This can lead to wasted time and resources for the researchers. In terms of existing tools, *Sona Systems* enables participants to choose from a set of times provided by the researcher (Sauter et al., 2020) but does not minimize or maximize the number of appointments to optimize attendance, while *Doodle* allows researchers to specify attendee maximums but lacks features to set minimums or to provide multiple sets of appointments. *Calendly* and *Microsoft Teams Scheduling Assistant* offer basic scheduling functions but do not support these more advanced features. Table 1 provides an overview of current platforms and their capabilities and highlights how effective Minimax, the algorithm underlying *Optimeet*, is in addressing these requirements.

The inefficiencies around group scheduling can have profound impacts on the associated research. For instance, Mao et al. (2016) described a lengthy process involving MTurk in which participants completed pre-screening questionnaires and stated their availability for specific dates. Study sessions were then scheduled according to popularity and participant preferences but encountered significant challenges, including insufficient attendance (not all invited participants attended), delayed start times, and group dropout rates that ranged from 4.5% to 14.2%. When participants sign up for appointments that are later deemed infeasible, cancelled, and prompt a request to reschedule, it can frustrate them and increase their likelihood of disengaging from the study altogether (Holdsworth et al., 2019; Strathmann & Hay, 2009; Tait et al., 1997). Thus, while appearing pragmatic at face value, self-selection of singular appointments limits the information available to researchers and constrains their ability to accommodate at least a portion of their sample, leading to a laborious allocation process that can impair participant retention.

### Optimizing group allocations across appointments

**Fig. 1:**
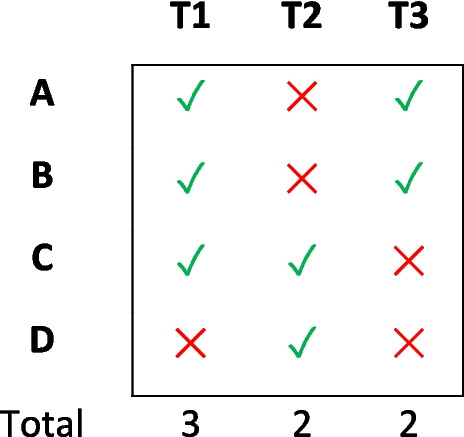
An example appointment allocation scenario. *Note.* Letters A, B, C, and D symbolize participants, and T1, T2, and T3 represent available time slots. Ticks denote time slots for which participants have indicated their availability, while crosses signify their unavailability

An allocation system that optimizes groupings across appointments would mitigate the problems described above. Consider a hypothetical scenario in which a researcher needs to recruit dyads or triads, and four participants (A, B, C, D) are interested in taking part (see Fig. 1). The researcher provides three time slots for the participants to choose from (T1, T2, T3), and the participants’ choices are as follows: A: T1, T3; B: T1, T3; C: T1, T2; D: T2. In this case, the most popular time slot (T1) has three sign-ups. However, because of the required group sizes, choosing to run T1 on this basis would only provide the researcher with

one group, while the other time slots would be rendered infeasible because neither would furnish at least two eligible people.

Suppose we had an algorithm that proposed a context-sensitive solution, whereby participants A and B are allocated to T3, and participants C and D to T2. This set-based solution optimizes participant use by allocating all four into two distinct dyads. Unfortunately, in larger samples, the combinatorial complexity grows, increasing the likelihood of a researcher making a manual allocation that is inefficient. Prioritizing the most popular time slots without considering the rest of the sample can result in inefficient allocations, which can lead to underpowered group research and wasted resources.

Here, we present a practical solution to address the needs of researchers when allocating individuals into groups for single appointments. Specifically, *Optimeet* is a web application for group-based scheduling that is powered by heuristic decision rules and allows researchers to specify their requirements and improve their appointment schedules. The solution includes a how-to guide and short tutorial to support researchers, as well as open-source code and documentation to support transparency and reproducibility.

We describe the decision algorithms that underpin *Optimeet*, and empirically validate the solutions it proposes. Specifically, we ask (1) what circumstances cause sets of algorithms to perform well, and (2) which rules fare best. We go on to present evaluation metrics that enable researchers to assess the quality of an appointment allocation within the context of their specific study requirements, and we validate a set of heuristics for making allocation decisions and apply these to a variety of different research-scenario simulations to compare their performance across contexts. Finally, we verify the performance of these strategies using a large real-world (human) dataset.

### *Optimeet*: A web application for group-based scheduling

**Fig. 2:**
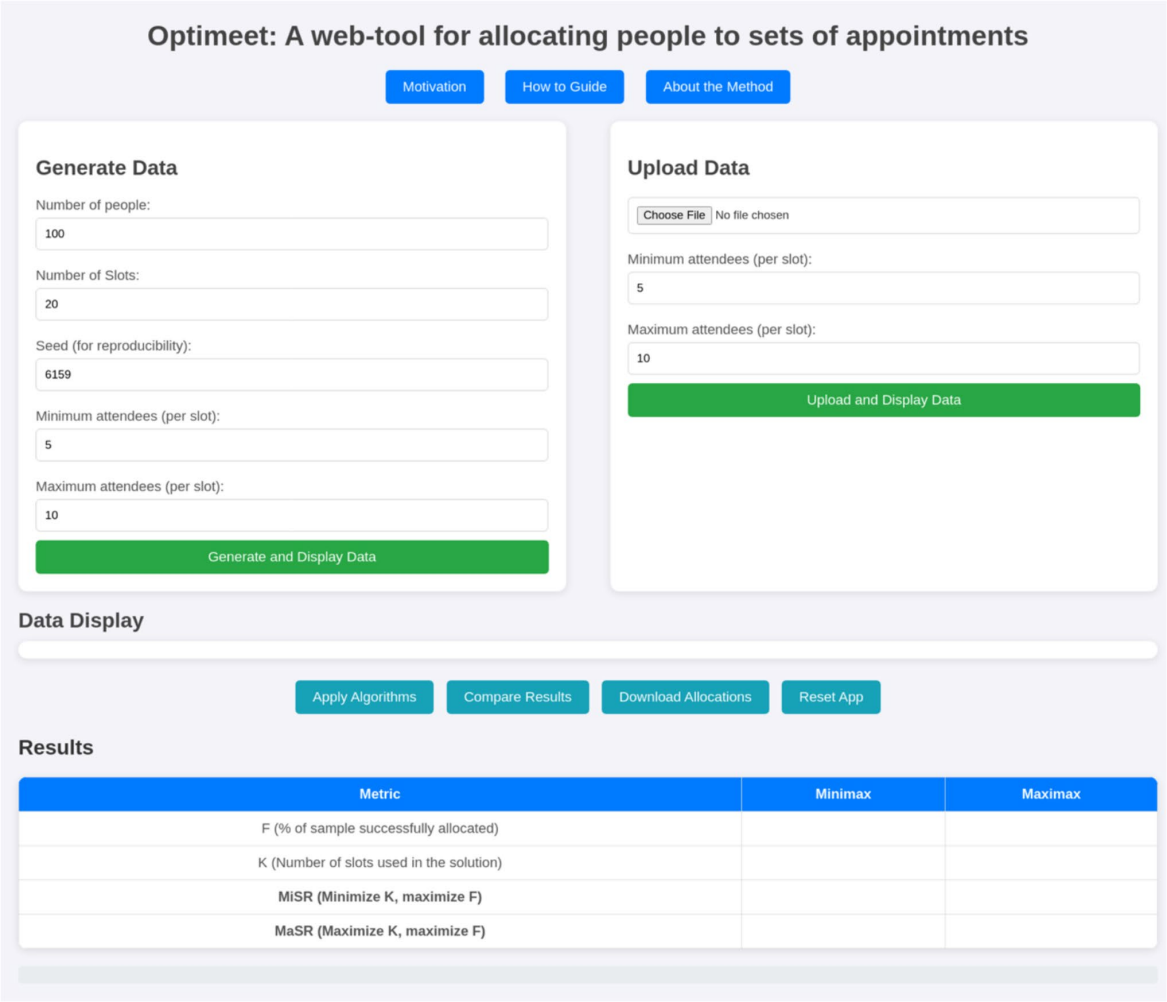
Screenshot of the *Optimeet* web interface. *Note.* The three buttons at the top provide essential background information, as well as a usage manual. Users can use the app to generate artificial data to evaluate the allocation algorithms (on the left side) or upload their own data (right side). The “How-to Guide” links to a further document that outlines how to format data for use in the tool. The 'Data Display' section provides visualization of the information to which the algorithms will be applied, allowing users to verify its format. Below this are the tool's main controls, with the results presented in tabular form beneath. The app can be accessed here, with source code available here

*Optimeet* is a web-based application that allows users to specify their requirements and optimize group appointment schedules (see Fig. 2). It is designed to help researchers allocate individuals to group appointments efficiently by using heuristics—decision-making shortcuts that produce efficient, if not necessarily optimal, solutions (Gigerenzer & Gaissmaier, 2011; Pearl, 1984). Heuristics are especially useful in scheduling problems, where identifying an optimal solution is often impractical owing to the complexity involved. Heuristics allow researchers to generate workable solutions quickly, even in complex or resource-constrained environments (Burke et al., 2003). To this end, *Optimeet* implements two empirically validated heuristics: “Maximax” and “Minimax.” Maximax is an optimistic popularity-first heuristic that iterates over time slots in order of their popularity, assigning as many available participants to a slot as its capacity allows before moving on to the next. It mirrors the most intuitive, first-come-first-filled approach. Minimax is a more pessimistic scarcity-first heuristic that iterates over time slots in reverse order of their popularity, assigning participants to the least popular slot that can be filled to a minimally sufficient level before moving on to the next. It aims for a more even distribution of participants by using the least desirable time slots. Both heuristics are core to *Optimeet*’s functionality and have been evaluated through simulations, described in later sections of this paper.

*Optimeet* allows users to upload a file of comma-separated values (CSV) representing participants’ availability data and to specify the constraints for group sizes. It uses these inputs to generate schedules intended to reduce resource wastage and improve attendance. Its key features include the following:

- Data input and visualization: Users can generate sample data or upload their own CSV files containing participant availabilities. *Optimeet* displays uploaded data for verification purposes.
- Algorithm application: Users can apply two of the best-performing scheduling algorithms (Minimax and Maximax) to optimize group allocations. *Optimeet* provides a comparison of the results according to key performance metrics.
- Customization and flexibility: Users can specify parameters such as the number of participants, number of time slots, and minimum and maximum attendees per slot. To support effective use, we have provided sample instructions for participants that researchers can use to encourage them only to select time slots they can realistically and comfortably attend (see: https://osf.io/z8gbs). Clear communication at this stage is crucial in reducing no-shows and improving allocation reliability.
- Output and accessibility: Users can download the generated schedules for ongoing use. A detailed how-to guide and video tutorial have also been created: https://osf.io/q9jy8/?view_only=fb8a226a336c49ddbea92e85e35525e5
- Open-source code: Users can tailor the application to their own needs or expand its functionality freely by modifying, extending, and distributing the code that underlies *Optimeet*. Further, because *Optimeet* is fully open-source and not dependent on proprietary platforms, it is less vulnerable to associated service disruptions and system changes. It is available in both Python and JavaScript, enhancing accessibility and ease of maintenance.

In the following sections, we document the decision algorithms that underpin *Optimeet*, and validate its output empirically.

## Experiment 1: Simulation

### Method

We aimed to build *Optimeet* to allocate people to context-sensitive sets of appointments. This raised several challenges, such as how to describe the problem, what solutions might be possible, and what methodology should be used to evaluate these. Building a feasible framework for addressing such complex challenges requires them to be framed in computational terms, because trial-and-error investigations would prove immensely costly. To make the exercise tractable, we formalized plausible simulations of human behavior to generate artificial scheduling data. We used this data to sense-check the algorithms powering *Optimeet*'s allocations and thereby avoid expending research funds better spent on validating the resulting outputs.

Once these elements of the research were established, the examination and precise comparison of different algorithms could be performed without the involvement of real-life participants, conserving resources. Following the simulation work, the solutions found to be most effective were subjected to separate confirmatory studies with real participants to verify the adequate alignment of problem formulations and solutions. Essentially, we express human availability decisions as a probabilistic process and then simulate different sample-level outcomes for various research contexts, such as the level of interest in the study and the number of possible appointments. The mathematical details of these simulation procedures are outlined in Appendix A.

#### Heuristics

A small set of simple, parameter-free heuristics can facilitate allocation decisions from simulated sets of participant choices. Post-allocation, constraints are upheld by de-allocating all individuals assigned to time slots for which attendance falls below the minimum required (*M*_min_) or by indiscriminately removing as many as needed to ensure the maximum capacity (*M*_max_) is not exceeded.

**Fig. 3:**
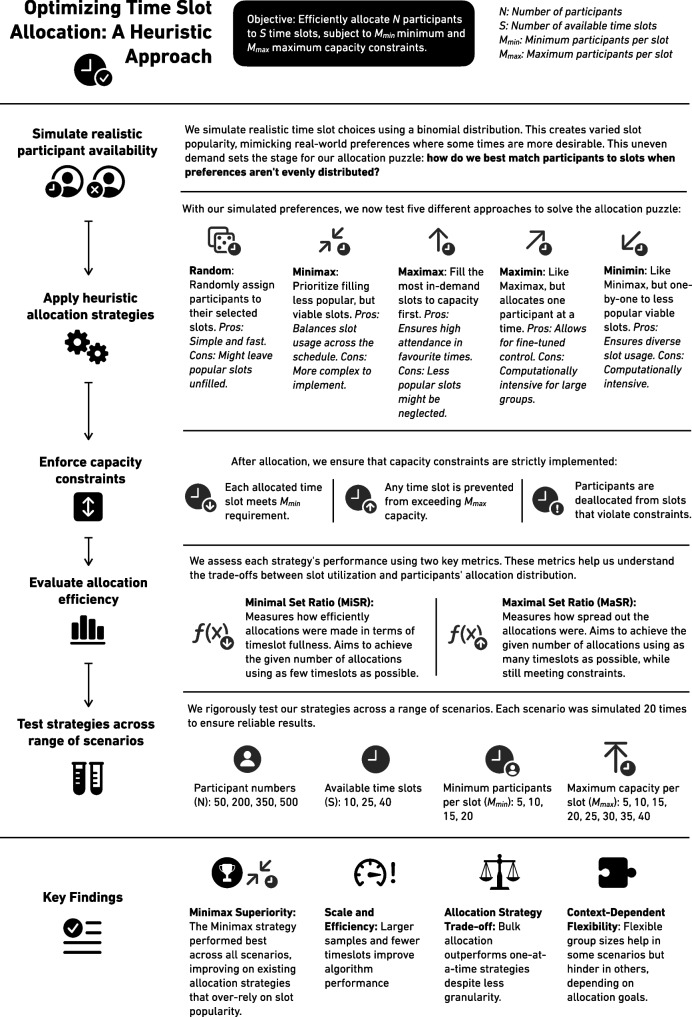
Heuristic approach to time-slot allocation. *Note:* This infographic outlines key methods around our four-step process for optimizing participant allocation to time slots: simulation, strategy application, constraint enforcement, and efficiency evaluation. Five heuristic strategies are compared and key findings are summarized

We chose this strategy because of the potential complexity of the combinatorial optimization problem and the cost-effectiveness of heuristics as algorithmic tools for intuitive initial explorations of computational issues. The use of heuristics is compelling because of their simplicity of implementation and their ability to provide satisfactory solutions with minimal computation. The utilization of simple decision rules allows most of the combinatorial space to be ignored, significantly reducing the computational burden necessary to reach a satisfactory solution. Their drawback is that simplified models cannot guarantee the ability to consistently identify the optimal solution and thus run the risk of underfitting the problem. This can be mitigated in part by constructing a set of heuristic models that enable precise performance comparisons between them, thereby facilitating judgments of their relative quality. Moreover, if such algorithms are nested—one being an extended version of another—this comparative exercise precisely disambiguates the magnitude of any potential benefit provided by the additional processing steps. The approach is summarized in Fig. 3.

We developed five heuristics, one being the Maximax heuristic, which aims to fill the most popular time slots to their capacity first. It starts by determining which time slots are most popular among the unallocated participants, then filters out those that have already reached their maximum capacity. In each iteration, the most popular time slot that still has capacity is selected, and as many participants as possible are allocated to this target time slot as its capacity allows. The process is repeated until all participants have been allocated.

The Minimax heuristic attempts to ensure an even distribution of participants by focusing on filling time slots with the lowest popularity that still meet the viable minimum (*M*_min_). It begins by identifying the least popular time slot that meets the minimum popularity and has not reached capacity, and participants are allocated to this time slot as its capacity allows or until no more participants have expressed a preference for it. Again, the process is repeated until all participants have been allocated.

The “Maximin” and “Minimin” heuristics are almost identical to Maximax and Minimax, respectively, the only difference being that they make just one participant allocation in each iteration. Thus, they require many more iterations before all allocations have been made. Finally, the “Random” heuristic simply randomly allocates each individual in turn to one of the slots they have selected. (For the exact implementation of these algorithms in Python code, please refer to *algos.py*, which has been uploaded to the OSF: https://osf.io/q9jy8/.)

The Random heuristic serves as a baseline model, and any algorithm that performs less well is deemed subpar; those performing better are regarded as operating above the level of pure chance. Maximax can be viewed as an iterative extension of existing availability-based scheduling tools capable of set-based solutions, such as Doodle polls. Conversely, in this context, Minimax represents an entirely novel strategy and is based on the idea that popular time slots can be filled with relative ease, even as a growing proportion of the sample is allocated, and one should, therefore, seek instead to prioritize allocations to less popular but still viable time slots. Maximin and Minimin can be seen as more computationally intensive variations of Maximax and Minimax, respectively, trading efficiency for greater precision in allocation decisions.

#### Simulation procedure

The heuristics were assessed across a range of research scenarios, including verying minimum and maximum attendance requirements (Mmin, and Mmax respectively), sample sizes (|*N*|), and time-slot set sizes (|*S*|). This test grid included scenarios that demanded precise group sizes and those permitting more flexibility in this, as well as scenarios in which demand outweighed capacity or capacity exceeded the number of people in the sample. To reflect realistic research environments, the parameter values were selected on the basis of prior empirical research on group dynamics and scheduling (e.g., Mao et al., 2016; Ogungbamila et al., 2010), which used comparable group sizes, attendance constraints, and coordination demands to those modeled in our simulations. These informed our choices around group size, time-slot availability, and minimum/maximum group capacities. The test grid was limited to combinations of the following values:

$$\begin{array}{l}|N| \in \{50, 200, 350, 500\}\\ |S| \in \{10, 25, 40\}\\ \begin{array}{l}{M}_{\text{min}} \in \{5, 10, 15, 20\}\\ {M}_{\text{max}} \in \{5, 10, 15, 20, 25, 30, 35, 40\}\end{array}\end{array}$$

Any combinations in which *M*_min_ exceeded *M*_max_ were omitted from the grid because of their practical impossibility. Because the generation of availability selections for sets of simulated participants employed random number generation, we repeated each simulation multiple times to improve the reliability of our observations. To generate random numbers, we employed a systematic approach: each independent simulation run used a sequential integer as its seed. The first simulation was initiated with a seed value of 1, and each subsequent run incremented this by 1. This ensured that every simulation had a distinct and reproducible starting point for the random number generator, avoiding duplication while supporting transparency and replicability. The final test grid included 312 scenarios, repeated for 20 random instantiations, totaling 6,240 unique simulations.

Given a set of parameter values from the grid, a set of participants was generated using the values for |*N*| and |*S*|, together with a seed value (effectively, an arbitrary integer). To induce variation among participants and time-slot popularity, each participant’s availability was generated from a binomial distribution, rather than assuming equal probability across time slots. This ensured that some time slots were more popular than others, better reflecting real-life scheduling situations in which availability is not uniformly distributed. Each of the five heuristics was then applied in turn to the sample to generate a solution. Subsequently, the constraint values (*M*_min_, *M*_max_) were imposed on these solutions to ensure that only valid allocations remained: if a slot was overfilled or underfilled, associated participants were reassigned as *“*Unassigned” to maintain slot constraints. For example, if a solution had assigned 25 people to a slot with a maximum capacity of 20, five people were reassigned in this way. Finally, relevant metrics for the finalized solutions were recorded, such as the grid parameters, the number of time slots used, and the proportion of successful allocations. This procedure was repeated for every set of parameters and seed values.

### Results

#### Data processing

The final dataset consisted of the summary statistics for each of the five algorithmic solutions (following constraint enforcement) when applied to the 6,240 simulations, producing 31,200 solutions in total. Basic information about each of these was recorded, including (1) the sample size, (2) the number of available time slots, (3) the minimum and maximum capacities of time slots, and (4) the numeric seed used to randomly generate the selections of simulated participants. In addition, binary variables captured whether the simulation required exact groups (*FlexibleGroups*) or featured excess capacity of time slots in relation to the sample size (*ExcessCapacity*), defined thus:

$$\begin{array}{l}Flexible\ Groups=\left\{\begin{array}{lc}0\ if\ {M}_{min}= {M}_{max} \\ 1\ if\ {M}_{min}\ne {M}_{max} \end{array}\right.\\Excess\ Capacity=\left\{\begin{array}{lc}0\ if\ {M}_{max} \times \left|S\right|\le \left|N\right| \\ 1\ if\ {M}_{max}\times \left|S\right|>\left|N\right| \} \end{array}\right. \end{array}$$

Moreover, the final dataset included solution-specific variables, such as (1) the specific algorithm applied to the simulated selection, (2) the proportion of the sample that was successfully allocated (*F*), (3) the number of time slots (*K*) the solution featured, and (4) “minimal set ratio” (*MiSR)* and “maximal set ratio” (*MaSR*) scores, defined in detail in Appendix A. Intuitively, the *MiSR* score reflects the degree to which a solution was able to allocate the sample to as few time slots as possible (i.e., fewer, bigger groups), while *MaSR* scores capture the extent to which allocation maximized both the proportions of successful allocations and of time slots used in the solution (i.e., more, smaller groups). As such, a high *MiSR* is desirable in resource-constrained settings, while a high *MaSR* is preferable when statistical calculations are to be performed at the group level.

#### Exploratory analysis: Differences among simulation settings and algorithms

**Fig. 4:**
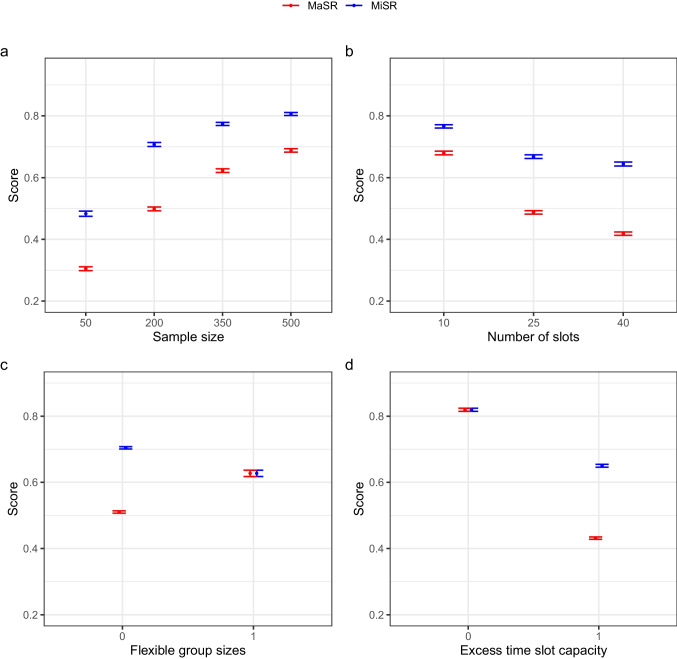
Average minimal set ratio (MiSR) and maximal set ratio (MaSR) scores across different study conditions. *Note:* Average minimal set ratio (*MiSR*) and maximal set ratio (*MaSR*) scores, with 95% confidence intervals, achieved across different study conditions: **a** per sample size; **b** per number of slots; **c** group-size specificity; **d** excess time-slot capacity

Across the algorithms, there was a noticeable trend for higher *MiSR* and *MaSR* scores for larger samples and smaller numbers of slots. These are graphically represented in Fig. 4a and b. Further, we can observe divergent trends between the score types for the two distinct values of *FlexibleGroups*. In the case of *MiSR* scores, having a range of capacity for time slots (*FlexibleGroups* = 10) proves detrimental to allocation quality (mean [*M*] = 0.63, standard deviation [*SD*] = 0.34, in contrast to *M* = 0.70, *SD* = 0.31), but proves beneficial for *MaSR* scores (*M* = 0.63, *SD* = 0.34, compared to *M* = 0.51, *SD* = 0.30). This observation is further illustrated in Fig. 4c. Meanwhile, it seems that the presence of excess time-slot capacity (*ExcessCapacity = *1) in relation to sample size is detrimental to both scores (*MiSR*: *M* = 0.65, *SD* = 0.33, compared to *M* = 0.82, *SD* = 0.21; *MaSR*: *M* = 0.43, *SD* = 0.28, compared to *M* = 0.82, *SD* = 0.21), as illustrated in Fig. 4d.

To test whether these differences between simulation conditions were supported statistically, we computed two analyses of variance (ANOVAs; one for each metric) and followed up with post hoc contrast tests. Both models included the predictors relating to sample size, number of available slots, exact group sizes, and excess time-slot capacity, while the *MiSR* and *MaSR* metrics were rendered as the outcome variables. All effects in the model for *MiSR* were found to be statistically significant, for sample size (*F*(3, 31192) = 1549.70, *p* < 0.001, η^2^ = 0.13), number of slots (*F*(2, 31192) = 292.62, *p* < 0.001, η^2^ = 0.02), *FlexibleGroups* (*F*(1, 31192) = 314.15, *p* < 0.001, η^2^ = 0.00), and *ExcessCapacity* (*F*(1, 31192) = 14.62, *p* < 0.001, η^2^ = 0.00). Likewise, the *MaSR*-based model showed a significant effect for all predictors: sample size (*F*(3, 31192) = 1726.89, *p* < 0.001, η^2^ = 0.14), number of slots (*F*(2, 31192) = 797.38, *p* < 0.001, η^2^ = 0.05), *FlexibleGroups* (*F*(1, 31192) = 228.10, *p* < 0.001, η^2^ = 0.00), and *ExcessCapacity* (*F*(1, 31192) = 1786.88, *p* < 0.001, η^2^ = 0.05). It should be noted that many of these effects are rather small, as a result of summarizing across five algorithms with varying outcome patterns.

Pairwise contrast tests were computed in order to evaluate the effect that different conditions of the variables supported by the ANOVA outputs had on score values. The tests independently compared each level of the predictor variables against one another. This post hoc analysis aimed to detect effects that showed divergent or noteworthy patterns with effect sizes of at least *d* ≥ 0.2. This threshold was chosen to protect against the identification of effects so minute as to be of no practical relevance, and for its conventional association with “small” effects. All pairwise comparisons emerging from both ANOVA models were found to be statistically significant, though not all with *d* > 0.2. The full set of contrast test results can be found in Tables S1 and S2 of Appendix B.

**Fig. 5:**
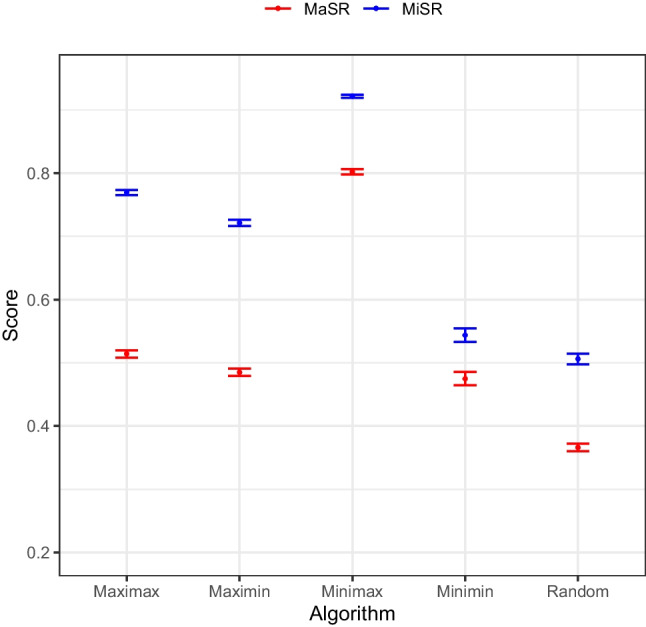
Average minimal set ratio (*MiSR*) and maximal set ratio (*MaSR*) scores for different algorithms, with 95% confidence intervals. *Note:* Average minimal set ratio (MiSR) and maximal set ratio (MaSR) scores with 95% confidence intervals

We also observed sizeable differences between the algorithms, although the trends appear largely consistent across score types. Across both scores, the baseline Random heuristic performed worst across the range of conditions (*MiSR*: *M* = 0.51, *SD* = 0.34; *MaSR*: *M* = 0.37, *SD* = 0.25) and the Minimax algorithm performed best (*MiSR*: *M* = 0.92, *SD* = 0.09; *MaSR*: *M* = 0.80, *SD* = 0.16). The scores for the entire set of algorithms are represented in Fig. 5.

We computed two more ANOVAs with post hoc contrast tests to elucidate whether there was statistical evidence of differences between algorithms across various conditions for our two metrics. It should be noted that our exploratory analyses to identify interactions between algorithms and specific combinations of experimental conditions proved redundant, owing to the dominance of one algorithm throughout most of the scenarios and its equivalence with other algorithms in the remainder. Consequently, the third and fourth ANOVA models only included, respectively, the algorithm applied as a predictor for *MiSR* or *MaSR*; the effect was found to be statistically significant in both instances, respectively: *F*(4, 31195) = 2404.50, *p* < 0.001, η^2^ = 0.24; *F*(4, 31195) = 2207.20, *p* < 0.001, η^2^ = 0.22.

We also ran pairwise contrast tests to elucidate which algorithms differed from each other in terms of their mean scores. All pairwise comparisons of algorithm performance were statistically significant for the *MiSR* case. This was also true for the *MaSR* case with the exception of the comparison between Maximin and Minimin. Some comparisons involving Minimin and/or Maximin did not exceed our *d* > 0.2 threshold. The full contrast test results are available in Tables S3 and S4 of Appendix B.

### Discussion

In summary, the results of Experiment 1 indicated those situations for which the set of candidate algorithms fares well as a whole, as well as highlighting performance differences between the algorithms. In general, the algorithms performed better with larger samples and fewer time slots. Limiting time slots increases the likelihood of forming full groups, thereby improving allocation efficiency. However, the influences of group size and appointment capacity relative to sample size appear to be contingent on specific optimization goals. Overall, where feasible, we recommend that researchers aim to recruit as many participants as possible and limit the number of time slots offered. When this was the goal, all of our heuristics improved on the Random baseline, but the Minimax algorithm consistently provided the best performance. These initial results established a strong foundation for further validation, which we explored in Experiment 2 by testing the algorithms with real participants to determine whether the simulation's findings hold in practice.

## Experiment 2: Real-world/human study

### Method

Experiment 2 aimed to validate empirically whether the differences observed between heuristics in silico are also observed with human participants. Because we argue that, for the purpose of our algorithm development, our simulation procedure is sufficient to match qualitatively the responses generated by humans, we hypothesized that all of the effects observed in the simulation will be replicated in a human sample. Thus, participants were recruited and asked to indicate their availability for a (hypothetical) study. Compared to Experiment 1, this preregistered follow-up experiment utilized a simplified design because many intermediary conditions had been found not to be informative in distinguishing between or among algorithms. The experiment received ethical approval from the Social Science Research Ethics Committee (Reference: 0455-396) at the University of Bath and was conducted in accordance with guidelines provided by the British Psychological Association (BPS).

#### Participants

We recruited 5,289 unique participants through Prolific, each compensated with 0.30 GBP (~0.40 USD). Prolific collects demographic information about participants, but no personally identifying information was collected, with all responses anonymized and participants identifiable only by a unique Prolific identifier. The only requirements for participation were an age of 18 or above and fluency in English. Information about the evaluation of an appointments system was given to participants, who gave their informed consent prior to commencement of the task. The final sample consisted of 49.6% self-identifying as female, 50.1% as male, and 0.3% as “Other”; the mean age was 36 years (*SD* = 12.83), and 75.1% of participants identified as “White,” 15.1% as “Black,” 4.4% as “Asian,” 3.6% as “Mixed,” and 1.3% as “Other” (0.5% did not identify).

For transparency, it should be noted that we preregistered the collection of 5000 unique participants but ended up receiving data for 5,289, despite Prolific not allowing more than one submission per person by default. The most likely reason for this discrepancy is that our study was both very brief and lucrative, and therefore the Prolific system probably received and accepted a higher level of simultaneous submissions than expected, leading to a greater-than-expected sample size.

#### Design

Experiment 2 involved a between-subjects design. Participants were randomly assigned to one of two conditions: (1) a low-slots condition wherein participants indicated their availability for any of 10 time slots, and (2) a high-slots conditions in which the number of time slots offered was 40.

At the allocation level, this experiment included a further set of conditions pertaining to the sample size (two levels: 50 or 500 people), the minimum time-slot attendance requirement (four levels: 5, 10, 15, or 20 people), the maximum time-slot capacity (eight levels: 5, 10, 15, 20, 25, 30, 35, or 40 people), and the form of algorithm applied (five variants: Random, Maximax, Minimax, Maximin, or Minimin). Allocations were performed on nonparametrically bootstrapped samples (i.e., resampling with replacement) from the participant pool. To increase the reliability of outcomes and account for sampling variability, each unique combination of conditions was repeated across 100 seeds of bootstrapped samples. Random seeds were assigned using a simple incremental procedure in which the first run used a seed value of 1 and was increased by 1 in each subsequent run. The number of seeds necessary was calculated on the basis of a power analysis for replicating effects of size *d* ≥ 0.2. Specific details can be found on our OSF page (see: https://osf.io/gbzn).

#### Materials

The survey consisted of a matrix-style set of checkboxes (see the corresponding screenshots on the OSF page under Experiment 2: https://osf.io/q9jy8/) by which participants could indicate their availability for various time slots, spanning several days and times. Days from Wednesday through Sunday were always included, together with 9 a.m. and 6 p.m. time slots on each day (i.e., 10 time slots in total); the high-slots condition offered additional daily time slots of 10 a.m., 11 a.m., 1 p.m., 2 p.m., 3 p.m., and 4 p.m. (i.e., 40 in total). Following completion of this matrix, participants were asked to rate the convenience of the selection procedure on a scale from 0 to 100, where 0 represented “Not convenient at all,” and 100 indicated “Very convenient.”

#### Procedure

Having signed up through the Prolific platform, participants completed the experimental task using an online survey hosted on QuestionPro. Participants were provided with an information sheet and informed consent was obtained. In order to increase the validity of responses, we informed participants that we were asking about their availability in relation to a potential follow-up study involving a payment. The online survey concluded with a debrief section in which participants were informed that there would be no follow-up study and that this information had been included to elicit more realistic responses.

Once all responses had been collected, the data was processed into two separate data frames according to the slot conditions. For each combination of constraint conditions (excluding combinations where the minimum attendee requirement exceeded the maximum time-slot capacity), the algorithms made allocations using nonparametrically bootstrapped samples drawn from these data frames. These allocations were repeated for 100 seeds because the bootstrapping employed random number generation. Metrics concerning the allocation and the underlying structure of the input information were recorded and served as the final data for the analysis. Both this processed dataset and the survey responses from participants have been uploaded to the OSF: https://osf.io/q9jy8/.

### Results

#### Confirmatory analysis

In Experiment 2, we sought first to validate the performance of the algorithms using data collected from people wishing to take part in a study. We hypothesized that all statistically significant effects reported in the post hoc analysis of Experiment 1 with *d* ≥ 0.2 would be replicated in a human sample, and found this to be the case for nearly all such effects.

The dataset derived from this experiment was identical in structure to that from Experiment 1, only differing in size because of the higher number of repetitions and the reduced set of conditions. In this case, five algorithms were applied to 100 random instantiations of 104 unique scenarios, meaning the data totaled 52,000 observations. This design difference was implemented on the basis of a power analysis (reported in the OSF: https://osf.io/q9jy8/) designed to test for the effects observed in Experiment 1.

The same structures utilized by the ANOVAs reported as part of Experiment 1 were applied to this data, testing whether the specific combinations of conditions meant to reflect a variety of research settings had differing effects on allocation success in this confirmatory study. All of the ANOVA models were statistically significant (*p* < 0.01), demonstrating that the key factors identified in Experiment 1—sample size, number of slots, *FlexibleGroups*, and *ExcessCapacity*—continued to have an impact on allocation success in this more real-world situation (see Appendix C for further details). These omnibus tests were followed by the same set of pairwise contrast tests described in Experiment 1. The full set of statistical test outcomes can be viewed in Tables S5 through S8 in Appendix C. All of the noteworthy effects of Experiment 1 were observed and were statistically significant with an effect size of *d* ≥ 0.2, the single exception being *FlexibleGroups* in the *MaSR* contrast test (see Table S6).

#### Exploratory analysis: Differences between simulated and human data

The target of our final, exploratory analysis was the level of alignment between the simulation procedure and real human behavior. This comparison was important in validating the assumptions underpinning Experiment 1 and assessing the extent to which simulation-based scheduling outcomes might be generalized to real-world scenarios. To this end, we employed a set of methodologies to (1) measure the level of alignment and (2) explore the source(s) of any deviations identified. We found that the simulations aligned well with our human-derived data, although people sometimes provided fewer indications of availability than we used in our simulations.

All bar one of the relevant effects seen in Experiment 1 were replicated in Experiment 2. There was, however, some notable variation in their size (Tables S5 through Table S8 in Appendix C provide more detail), which may be an artifact of the smaller grid of conditions used in Experiment 2. Alternatively, the simulation of behavior employed in Experiment 1 may differ systematically from real-world response patterns. In order to evaluate the difference between simulated and human behaviors more precisely, a further set of simulations was computed that mirrored the exact condition structure of Experiment 2.

To measure the alignment between simulated and real-world data, the source of an allocation (i.e., whether it originated from the simulation or real-world behavior) can be conceptualized as a rater, allowing for the use of inter-rater agreement measures to quantify the similarity of allocation patterns across condition-matched scenarios. Given the numeric nature of the *MiSR* and *MaSR* metrics, the most applicable method for this is the intraclass correlation coefficient (ICC). Computing the ICC between the situation-matched allocation scores that arose from the simulated data and that of real-world behaviors revealed a significant correlation, indicative of good, albeit not excellent, inter-rater reliability: *MiSR*: *ICC*(A,1) = 0.82, *F*(51999, 12367) = 10.00, *p* < 0.001; *MaSR*: *ICC*(A,1) = 0.84, *F*(51999, 12230) = 11.9, *p* < 0.001. Thus, the simulations employed in the first part of this study can be said to align well with real-world behaviors, although not perfectly so.

To disentangle what could be several potential sources of misalignment, interpretable machine-learning algorithms were applied to identify patterns predictive of the data source, analyzing the factors of highest importance to the model to indicate the source of the deviation(s). In other words, when treating the source of the data as a binary classification task, simpler machine-learning models whose parameters are interpretable can be used as tools for exploratory data analysis. Algorithmic families suited to this include decision trees, general additive models, and regression-based methods. We chose to focus on decision trees owing to the accessibility granted by interpreting the model as a graph. Specifically, we used a classification and regression tree (CART) method implemented in the *rpart* package in R (Therneau et al., 2013), which constructs binary trees on the basis of recursive partitioning. To ensure interpretability and prevent overfitting, the decision tree was limited to a maximum depth of seven. The model was evaluated using 10-fold cross validation with an 80/20 train–test split.

The overall accuracy of the classifier was 63.20% (95% CI: 62.54%, 63.86%; true positive rate [TPR] = 76.89%; true negative rate [TNR] = 49.51%). The prediction problem was balanced, and the real-world data was considered the positive class. The essence of the graph can be summarized as variables related to large numbers of time slots being indicative of the simulated data, especially when paired with high values of metrics indicative of allocation quality. This finding broadly aligns with the observation that better algorithms show an overconfidence bias, but also indicates that there may be differences between simulations and real-world behavior in the high-slots condition. Following this finding, the mean number of choices in the high-slots condition of the real-world sample was calculated and compared to the estimated mean of the simulated sample: they were, indeed, found to be notably different (simulation: *M* = 20.0, *SD = *3.16; real world: *M = *15.72, *SD = *10.76). Note that the estimated mean was used for the simulated sample because, to reduce memory usage, the simulations only recorded the performance metrics post-allocation; however, given our reproducible procedure, it was possible to analytically derive the population mean using the probability weights outlined in Appendix A.

These results suggest that simulations may modestly overestimate allocation quality in conditions that involve large numbers of time slots, a point worth considering when interpreting the findings of Experiment 1. While the overall trends in real-world behavior are replicated well in the simulation, researchers using *Optimeet* in such high-slot contexts should anticipate greater variability in the real world.

### Discussion

In summary, the results of Experiment 2 indicate that our simulations made reasonable assumptions about real-world participant availability selection: almost all of the effects that we observed in the simulation-based Experiment 1 were replicated. Our exploratory analysis quantified the good degree of alignment between our simulated and actual results and identified potential causes for the remaining misalignment. From a practical standpoint, the results confirm the benefits of employing a Minimax algorithm when the goal is to maximize the rate of participation while minimizing the number of appointments.

## Discussion of experiments

Using two experiments, we have demonstrated how computational techniques can improve the allocation of individuals into groups for group-based studies. Experiment 1 simulated time-slot selection behavior to test the performance of five heuristics under varying conditions, including the number of selectable time slots, preferred group sizes, and available appointment allocations. The best performance was associated with a Minimax algorithm, which prioritizes allocations to the least popular time slots that still fulfill the maximum attendance criteria. Experiment 2 validated empirically that the differences between heuristics observed in silico are replicated with real-world human participants, although the latter selected fewer time slots than predicted. An exploratory analysis indicated that there was a slight overconfidence bias in the application of the algorithm as a result.

In both experiments, larger samples and fewer time slots increased the likelihood of high-quality allocations, because the algorithms had more participants to work with and fewer selections to distribute. Conversely, smaller samples, a greater number of time slots, and excess time-slot capacity relative to participants made high-quality allocation less likely. For “Min-set” allocations, which focus on as few, maximally filled time slots as possible (see Appendix A for more detail), flexible group sizes complicated the process, because such variability increases the combinatorial complexity. However, for “Max-set” allocations, which seek to maximize viable groups by restricting group sizes (again, see Appendix A), flexible group sizes were advantageous, because they reduced the burden of slot-filling constraints, resulting in relatively better allocations. Overall, across both simulated and real-world samples, allocation quality metrics indicated that reasonable solutions are more readily identified for the Min-set case than for the Max-set one.

In Experiment 1, the Minimax algorithm emerged as the most effective across all conditions, outperforming the Maximax heuristic, which relied more heavily on slot popularity. Almost all of the effects observed in Experiment 1 were replicated with the human subjects of Experiment 2, suggesting that our simulations align well with real-world behaviors. Further exploratory analysis confirmed a good level of alignment, with only minor deviations. Specifically, the human sample was found to select fewer time slots in the high-slots condition than random chance selections would have predicted. Extant research on participants' biases and decision-making heuristics could explain this finding. That is, humans do not make rational decisions in the same way that an algorithm might and algorithms may not, therefore, fully capture such reasoning. As such, Experiment 2 did not capture the multitude of heuristics that participants might employ when making selections, which include loss aversion (e.g., avoiding time slots that could conflict with future opportunities; Tversky & Kahneman, 1992), preference falsification (e.g., avoiding less desirable time slots for which they were actually available; Kuran, 1987, 1998), satisficing (e.g., selecting good-enough options rather than optimal ones; Artinger et al., 2022; Simon, 1956), and decision fatigue (e.g., reducing choices when faced with many options; Baumeister et al., 1998).

The findings of Experiments 1 and 2 suggest that practitioners interested in utilizing the algorithms presented here should consider carefully how their expected sample size relates to the number of time slots they are looking to fill, because the algorithms perform best when there are more willing participants than capacity (i.e., number of time slots multiplied by the maximum number of attendees per slot) allows. Recruiting in waves may be one method that ensures that the number of people looking to participate exceeds slot capacity while still maximizing the total sample size. A default opt-in approach—in which participants are assumed to be available for all time slots and must explicitly opt out of unsuitable ones—could simplify the allocation process (Tentori et al., 2022; Yan & Yates, 2019). In the following sections, we discuss the wider implications of our findings with respect to the *Optimeet* tool, as well as directions for ongoing research.

## General discussion

The present research aimed to address a widespread methodological challenge in the behavioral and social sciences concerning the allocation of individuals into groups for research purposes. By incorporating our best-performing algorithms, Minimax and Maximax, *Optimeet* allows researchers to quickly gather details of participants’ availability and efficiently allocate the participants in a manner likely to maximize attendance. Of course, this still does not guarantee that attendance, but the method outlined here remains superior to current alternatives both in terms of efficiency and functionality. By helping researchers to more efficiently reach a desired or predetermined sample size, particularly in studies that require group-based coordination, *Optimeet* can support research across a wide range of subject areas and contexts. It may, therefore, help to reduce the risk of studies being underpowered because of participant dropout and scheduling inefficiencies, supporting more complete and reliable data collection. It can also support more complex research designs, such as those that require in-person coordination and interaction. Indeed, the challenge of coordinating teams and groups for repeated experimental testing is widely acknowledged (Benishek & Lazzara, 2019), and the need to explore more complex phenomena, alongside the need for methods that support this, has been highlighted in leading psychology journals that publish group work, including the *Journal of Personality and Social Psychology* (Kitayama, 2017) and the *Journal of Applied Psychology* (Eby, 2022).

Furthermore, *Optimeet* may help researchers to resist study designs chosen simply for their convenient fit with online participant recruitment platforms and, instead, embrace behavioral group-based phenomena in some of the more underexplored and challenging areas that combine online and offline behaviors (Smith et al., 2023), standing in sharp contrast to the short, micro-, or single-task experimental designs/crowdsourced activities that typically prevail in online-only studies (Sassenberg & Ditrich, 2019). In a similar vein, although online recruitment platforms can provide access to more geographically varied samples, participants tend to be technologically savvy and adept at undertaking research (Arguinis et al., 2020; Landers & Behrend, 2015; Paolacci & Chandler, 2014); the group-scheduling support that *Optimeet* provides may facilitate recruitment of those who are less familiar with research processes and/or the technologies that support them (e.g., those without computers, older adults).

In sum, *Optimeet* provides an efficient solution for organizing participants into groups and managing their attendance, particularly for complex and hybrid research designs. Our validation studies confirmed its effectiveness, demonstrating robust alignment between algorithmic models and participant behavior.

### Limitations and future directions

Despite the improved solution to maximizing group-based attendance that *Optimeet* provides, it has a number of limitations that could be addressed in future research. First, the underlying approach is best suited to organizing relatively simple meetings in which a group is required to meet only once. It cannot support more complex or longitudinal designs such as those that involve allocating individuals into groups that meet on multiple dates/times over a designated period, although *Optimeet* could be implemented repeatedly with the same group to facilitate meeting multiple times or allow for the dynamic updating of availability. Similarly, *Optimeet* does not allow researchers to specify demographics or other configurations that may be of interest when allocating individuals to groups (e.g., creating groups with a specific gender, age, or cultural balance).

Second, both *Optimeet* and our associated analyses have focused on participant availabilities that are static, interchangeable, and unchanging. Consequently, they do not account fully for certain participant behaviors that could influence the efficacy of meeting schedules. For instance, as noted earlier, participants may display a range of heuristics and biases when disclosing their availabilities: they may not disclose their complete availability (e.g., loss aversion, preference falsification), or they may strategically reveal only the times they prefer, intentionally withholding other possible but less desirable times (e.g., satisficing, anchoring). Participants may thus violate our modeling assumption that two time slots for which availability has been indicated are inherently interchangeable with respect to their likelihood of actual attendance. Although we do not believe that any platform could ever completely control for all individual differences, behaviors, or eventualities, future research could explore some of the reasons that people miss appointments for a given type of research, alongside more sophisticated tracking of preferences/dynamic updating of availabilities, and integrate the results into future iterations of *Optimeet*.

Third, to optimize overall group coverage, the Minimax algorithm deliberately distributes participants into those slots that are least popular but still viable. An underlying assumption is that participants only select times they are genuinely willing to attend. If slots are less popular because they are inherently less convenient, there is a risk that participants who mark such times out of uncertainty or flexibility may not follow through. This limitation is not unique to *Optimeet* but is important to acknowledge. We therefore encourage researchers using *Optimeet* to provide clear instructions within the availability survey (i.e., before data is submitted) to reduce the likelihood of attrition due to such participant-preference mismatches. The issue is likely to be exacerbated in studies with rigid group-size requirements (e.g., in which groups must contain an exact number of participants), where even minor levels of attrition can reduce session viability. Although *Optimeet* improves the initial allocation process, it cannot prevent dropouts. To maintain session integrity in such cases, researchers may wish to consider over-recruitment, the scheduling of buffer slots, or arranging backup participants.

Finally, there are a variety of technical limitations that could be addressed in future work in relation to the simulation procedures and the nature of the heuristic algorithms utilized. For instance, the heuristic algorithms were based primarily on popularity filtering; developing a wider range of algorithms could yield more powerful solutions than the ones employed here. A detailed overview of these considerations is provided in Appendix D. Future work could therefore refine the algorithms and simulations to better represent real-world behaviors and improve robustness across a greater diversity of settings.

### Beyond psychological science

Besides its methodological advances, *Optimeet*’s allocation algorithms also have significant implications for a variety of practice areas, one such being support of human resource management in recruitment or assessment centers. These often rely on a multitude of assessments, including group exercises, over the course of a day or a series of days (Ballantyne & Povah, 2016; Woodruffe, 2000). They require the effective scheduling of both job candidates and assessors (Breaugh, 2008; Leong, 2018), and an organization can incur vast expense in terms of staffing, time, resources, materials, and venues (Pattnaik & Padhi, 2021), while poor attendance can impede the evaluation of candidates (Karl et al., 2021) and the assessment of their future performance. More effective scheduling via our algorithms could help to support the efficiency, standardization, and consistency of processes that are important, both in terms of avoiding bias and ensuring fairness and equality throughout the recruitment process.

Our algorithms could also improve attendance of group appointments in healthcare (e.g., group therapy) and clinical trials, where missed and/or rescheduled appointments not only incur financial costs, but can exacerbate health problems, particularly for vulnerable populations (Dantas et al., 2018; Ellis et al., 2017; McQueenie et al., 2019). Most appointment scheduling research has focused on optimizing individual appointments (Ahmadi-Javid et al., 2017; Kuiper et al., 2023) and identifying factors that are associated with (non)attendance (Gupta & Denton, 2008; Wang et al., 2018); it has not offered any insight into how scheduling algorithms might be adapted for the purposes of group-based appointments. *Optimeet* provides a solution to this with a set of adaptable tools that can be readily applied to improve such scheduling. Our use of open-source code allows for customization to meet a variety of needs, which could broaden the impact of our work for a wide range of industries. For example, our algorithms could be combined with known predictors of nonattendance, such as prior missed appointments and lead time (Dantas et al., 2018), to further improve attendance, although chronic nonattendance involves complex dynamics, and relying solely on scheduling tools might exacerbate access inequalities for some patient groups (Lindsay et al., 2023).

### Conclusion

In summary, our findings demonstrate the utility of an online app that relies on simple heuristics for appointment allocation in research designs that are reliant on group appointments. The insights gained, along with the resources provided, have wider implications for the use of allocation heuristics in applied practice and provide important lessons for researchers when designing group studies. Aligning allocation strategies with research goals and ensuring that research designs maximize the applicability of these strategies show great potential to improve the resource efficiency of group-based research projects in the psychological and behavioral sciences.

## Supplementary Information

Below is the link to the electronic supplementary material.

Supplementary file1 (DOCX 54.1 KB)

## Data Availability

Data and materials are available in the Open Science Framework (OSF) [https://osf.io/q9jy8/].

## Footnotes

1 We note that there are multiple ways by which researchers may select sample sizes, and that these can be impacted by various factors, including resource constraints, planning for desired accuracy, and whether the research is exploratory or confirmatory (see, for instance, Lakens, 2022). Thus, while a priori power analyses reflect ideal practice in some contexts, they are not always reported, preregistered, or performed, and alternative approaches may be adopted in others.

## Code availability

Code is available in the Open Science Framework (OSF) [https://osf.io/q9jy8/].
