# Supplementary material for: Optimeet: A computational tool to enhance participant attendance in group research
Source: Behav Res Methods. 2025 Sep 17;57(10):289. doi: 10.3758/s13428-025-02745-9 (PMC12443933; doi:10.3758/s13428-025-02745-9)
Supplement: Supplementary file 1 — Supplementary file1 (DOCX 54.1 KB) [file 13428_2025_2745_MOESM1_ESM.docx]

**Appendix A: Mathematical Background**

In this section, we provide the mathematical details that underlie the essential parts of our simulation procedure and the derivation of our success metrics. We first discuss how, for the purposes of our algorithms, we abstracted human decision-making as a problem of sampling to arrive at simulated responses that are qualitatively indistinguishable from human ones. Second, we describe how, by working backward from the constraints of the problem, the success metrics with which we benchmarked our algorithms were derived.
**Simulating Time-Slot Selections**

When people indicate their availability for an appointment, their choices most often mirror their personal preferences. These preferences might include commitment to the workplace, obligation to family, cultural contexts, religious practices, and leisure pursuits. Given the broad array of potential influences, the availability of a specific person for a specific time slot can be viewed in probabilistic terms.

Thus, given a set of pre-specified time slots $S$ and a group of individuals $N$, there is a certain probability of $n\in N$ selecting $s\in S$. If $s_{i}$ is repurposed as a discrete outcome, it is possible to utilize random sampling to first determine a value representing the number of choices a hypothetical subject *k* would make ($C_{k}$, where $C_{k}$ is the set of choices of subject *k*), falling between 1 and $\left| S \right|$. Subsequently, the $\left| C_{k} \right|$ selections of the simulated participants can be drawn, without replacement, from the same set of integers. This procedure is sufficient to generate participant samples with selections that are uniformly distributed across time slots.

Simulating selections can be extended to include other discrete probability distributions by calculating normalized probability weights for outcomes under such distributions, and then sampling from the set of time-slot indices on the basis of these weights. For instance, if we consider the popularity of time slots to follow a binomial distribution, each $s_{i}$ can be contextualized as the number of successes in a binomial experiment with |*S*| trials (assuming a given success probability), and one can calculate the probabilities accordingly. In this research, selections were simulated on the sole basis of binomial distributions, with $\left| C_{k} \right|$ always uniformly drawn and the binomial trials success probability set to 50%. The rationale for this choice was that binomially distributed selections adequately represent scenarios in which certain time slots are more popular than others. This approach can be expressed more formally as follows. Let $\left| C_{n} \right|$ be the number of choices made by participant $n$. $\left| C_{n} \right|$ is chosen from at random. The $S_{i}:\left| C_{n} \right|\in RS_{i}$ probability weights are binomially distributed: $w\sim Binomial\left( \left| S \right|,0.5 \right)$. Excluding the event of zero successes, the probabilities are normalized: $w_{norm}=\left\{ \frac{w_{1}}{\sum w_{j}}, \frac{w_{2}}{\sum w_{j}}, \ldots, \frac{w_{n}}{\sum w_{j}} \right\}$such that $\sum_{i=1}^{n} w_{norm}=1$.

If $C_{n}$ denotes the set of choices for participant *n,* then$C_{n}=x_{1},x_{2},...,x_{c}\subseteq S_{i}$, where the probability of selecting $x_{p}$ is determined by the weight $w_{{norm}_{x_{p}}}$. This process is repeated for all $n\in N$, while maintaining the same set of probability weights.

**Optimal Allocation Strategies for Time Slots**

The structure of the problem at hand dictates that each time slot must fulfill a minimum attendance requirement to be feasible while also not exceeding a maximum capacity. When arranging sets of appointments, two potential outcomes can be considered: one scenario aims to maximize attendance with a minimally sized set of appointments, while the other aims to maximize attendance through a maximally sized set. To clarify, if hosting additional time slots incurs a significant cost, the optimal allocation must maximize the number of people allocated to time slots that meet capacity constraints while minimizing the number of time slots required to achieve this volume of attendees. On the other hand, if the goal is to maximize the number of completed group sessions, be it for statistical purposes or otherwise, then the optimal allocation should maximize the number of attendees and the number of constraint-satisfying time slots in use. Henceforth, these two cases will be referred to, respectively, as “Min-set” and “Max-set”.

Min-set can be interpreted using statistical modeling terminology, where the optimal allocation constitutes the model that maximizes model fit (the proportion of the sample allocated) while minimizing model complexity (the number of time slots used in the allocation). While these inputs to an objective function resemble those of information criteria and other statistical metrics (e.g., *R*^2^) that balance model fit and complexity, their application in this context is unsuitable owing to the unique interplay between the number of attendees and the number of time slots in an allocation. In the current context, model fit is inherently linearly dependent on model complexity owing to the capacity limits of time slots. For instance, given 50 people to allocate and a time-slot capacity of 10 people, no allocation could match all individuals to time slots with fewer than five such; a four-slot solution could only accommodate up to 80% of attendees, a three-slot solution 60% of attendees, and so on. This relationship can be described more formally when considering the properties of optimal allocation outcomes.

Thus, let $F$ denote the proportion of the sample allocated relative to the number that could potentially be allocated:

$$F =\left\{ \begin{aligned} \left| N_{A} \right|\div\left| N \right| if \left| S \right|\times M_{max}>\left| N \right| \\ \left| N_{A} \right|\div\left| S \right|\times M_{max} if \left| S \right|\times M_{max}\leq\left| N \right| \end{aligned} \right.$$

where $\left| N_{A} \right|$ is the number of people successfully allocated, $\left| N \right|$ is the sample size, $\left| S \right|$ is the number of time slots available for selection, and $M_{max}$ is the maximum member capacity of time slots.

The number of slots multiplied by the maximum membership count represents the total capacity of the appointments. If this total capacity exceeds the sample size, then it will be theoretically possible to allocate everyone in the sample, in which case $F$ would be the ratio of the number of people allocated to the sample size. However, if the capacity is less than or equal to the number of people to allocate, it will be impossible to allocate everyone in the sample without violating time-slot capacity constraints. In this case, it would only be possible to allocate as many people as the capacity allows, which is why $F$ must be the ratio of the number of people successfully allocated to the total appointment capacity.

Let $Q$ and $W$ represent the number of time slots required for a minimally or maximally sized set of time slots that maximize $F$, respectively:

$$Q =\left\{ \begin{aligned} \lceil\left| N \right|\div M_{max}\rceil if \left| S \right|\times M_{max}>\left| N \right| and \left( NmodM_{max}\geq M_{min} \right) \\ \lfloor\left| N \right|\div M_{max}\rfloor if \left| S \right|\times M_{max}>\left| N \right| and \left( NmodM_{max}<M_{min} \right) \\ \left| S \right| if \left| S \right|\times M_{min}\leq\left| N \right|\times M\_min \leq|N| \end{aligned} \right.$$

$$W=\left\{ \begin{aligned} \lfloor\left| N \right|\div M_{min}\rfloor if \left| S \right|\times M_{min}>\left| N \right| \\ \left| S \right| if \left| S \right|\times M_{min}\leq\left| N \right| \end{aligned} \right.$$

where $M_{min}$ represents the minimum attendee requirement for a time slot to be feasible.

Like $F$, the quantities $Q$ and $W$ depend on the relationship between the sample size and the total capacity of the time slots. For both quantities, if the sample size equals or exceeds the total capacity, then the optimal number of time slots is simply the given number of time slots, because this configuration will maximize the allocation of subjects. When total capacity exceeds the sample size, the calculation of the optimal number of time slots varies according to the set case.

For a minimized set size, the number of slots required to accommodate the entire sample is obtained by dividing the sample size by the maximum capacity. If the division’s remainder exceeds the minimum attendance requirement, the number is rounded up via a ceiling function; this prevents any subjects from missing an allocation when there is available space. If the division’s remainder falls below the minimum attendance requirement, a floor function is applied instead because the remaining participants would not fit in a time slot without exceeding capacity constraints.

Contrarily, for a maximized set size where there exists more capacity than attendees, the optimal number of slots is equal to the next lowest integer when the sample size is divided by the minimum attendance requirement for a time slot to be feasible. In this scenario, the division’s remainder does not need to be considered because it must, by definition, fall below the minimum attendance requirement.

Having established these quantities, one can effectively describe the dependency of fit values (*F*) on slot values (*K*). If we let $K$ be the number of time slots a proposed solution requires, then it is possible to show that $F$ must fall into the interval $\left[ \frac{K}{W},\frac{K}{Q} \right]$, so long as $K\leq Q$. In all other cases, $F$ will be in the interval $\left[ \frac{K}{W},1 \right]$. The lower bound of the interval signifies the value of $F$ where $K$ slots are filled minimally, while the upper bound equates the $F$-value where $K$ slots are filled to their maximum. Because $K$ can exceed $Q$ while $F$ cannot surpass 1, the upper bound of the interval is set to 1 when the former exceeds the latter.

Conversely, given $F$, one can infer that for a valid allocation, $K$ must be within the interval $\left[ floor\left( F\times Q \right),ceil\left( F\times W \right) \right]$. Because $F$ can only equal 1 when $K$ is either $Q$ or $W$, *K* must be within the inclusive interval of the downwards/upwards-rounded products of $F$-values, with the quantities representing maximal/minimal time-slot fill. In this scenario, the floor and ceiling functions ensure that any remainders captured in *F* but rounded out in *Q* or *W* are correctly incorporated. From this, it is possible to conclude that the relationship between *F* and *K* values is indeed linear, because the average value of these intervals, which can be interpreted as the outputs of the functions $f\left( K \right)$ and $g\left( K \right)$, exhibit the additivity and homogeneity properties of linear functions.

This linear relationship between model fit and model complexity contrasts starkly with statistical models in which the addition of parameters may yield non-linear benefits to model fit, usually in the form of diminishing returns. Owing to this difference, pre-existing information criteria yield perplexing outcomes. For example, the Akaike and Bayesian information criteria (Vrieze, 2012) behave in an overly conservative fashion, favoring allocations that underfit the sample, while *R*^2^ (Miles, 2005) does not sufficiently penalize overly complex solutions.

**Defining Objective Functions**

A purpose-built metric for allocation problems of this nature must balance a fit with the complexity of the solution with consideration of the constraints of the problem. To achieve this, one can examine the properties of the best-case solution and then compare the attributes of a proposed solution with this best-case scenario.

In the Min-set scenario, the optimal mathematical solution is the one that manages to allocate all individuals who could potentially be assigned ($F=1$) while utilizing the fewest time slots by maximally filling each one ($K=Q$). All that is required is a function to balance fit values with solution size values. In this study, both quantities were balanced with equal weighting. Because *F* values already lie within [0, 1], this proportion is multiplied by another ratio that quantifies how closely *K* matches *Q*. Thus, the “minimal set ratio” (*MiSR*) is:

$$MiSR=F\left( 1-\left| \left( 1-\frac{K}{Q} \right) \right| \right)$$

The part of the expression nested within the brackets ensures that any deviation of *K* from *Q*, either positive or negative, correctly reduces the ratio. This element is indifferent to the direction of the deviation, meaning that a solution utilizing, for example, two fewer time slots than deemed optimal is penalized to the same extent as a solution that incorporates two more time slots than necessary.

Conveniently, $K\leq W$ in all cases, meaning that for the Max-set scenario, the proportion of time slots used by a proposed solution compared to the optimal number of time slots is always simply the ratio of the two. Thus, the “maximal set ratio” (*MaSR*) is defined as:

$$MaSR=F\frac{K}{W}$$

Both metrics share a set of desirable properties. First, their values always lie between 0 and 1, inclusive, where 0 signifies the worst possible allocation quality and 1 symbolizes perfect allocation, rendering them easy to interpret. Second, because these metrics are the product of two fractions, large deviations are naturally punished heavily, while minor deviations are treated more leniently. Last, both metrics exhibit a slight relative preference for solutions that exhibit overfitting, because the formula is indifferent to the direction of set size deviations, but size and fit are equally weighted. This means that slightly more complex solutions (which are more likely to achieve a good fit) fare slightly better than slightly simpler ones. This propensity aligns with the common-sense logic that hosting a slightly larger set of slots with some excess capacity is preferable to a smaller set that will exclude a proportion of the sample.

**Appendix B: Experiment 1 Statistical Outcomes**

Here, we provide the statistical outcomes of the tests reported as part of Experiment 1 that, for brevity, were not discussed thoroughly in the main text. Tables S1 and S2 concern the evaluation of allocation difficulty according to each of the success metrics for different research settings, while Tables S3 and S4 compare the viability of algorithms according to these same success metrics.

**Table S1**

*Contrast test results for ANOVA 1.*

|  | Effect size | | | | | Cohen’s *d* | | |
| --- | --- | --- | --- | --- | --- | --- | --- | --- |
| Contrast | Estimate | SE | df | *t* ratio | *p* value | Cohen's *d* | Lower CL | Upper CL |
| *N*50/*N*200 | -0.22 | 0.00 | 31192 | -48.47 | <.0001 | -0.79 | -0.82 | -0.75 |
| *N*50/*N*350 | -0.28 | 0.00 | 31192 | -59.05 | <.0001 | -1.01 | -1.05 | -0.98 |
| *N*50/*N*500 | -0.31 | 0.01 | 31192 | -60.33 | <.0001 | -1.11 | -1.15 | -1.08 |
| *N*200/*N*350 | -0.06 | 0.00 | 31192 | -13.66 | <.0001 | -0.22 | -0.26 | -0.19 |
| *N*200/*N*500 | -0.09 | 0.00 | 31192 | -18.87 | <.0001 | -0.33 | -0.36 | -0.29 |
| *N*350/*N*500 | -0.03 | 0.00 | 31192 | -6.28 | <.0001 | -0.10 | -0.14 | -0.07 |
| *S*10/*S*25 | 0.09 | 0.00 | 31192 | 19.80 | <.0001 | 0.32 | 0.29 | 0.35 |
| *S*10/*S*40 | 0.11 | 0.00 | 31192 | 23.38 | <.0001 | 0.40 | 0.36 | 0.43 |
| *S*25/*S*40 | 0.02 | 0.00 | 31192 | 5.58 | <.0001 | 0.08 | 0.05 | 0.11 |
| *EG*0/*EG*1 | 0.08 | 0.00 | 31192 | 17.72 | <.0001 | 0.30 | 0.26 | 0.33 |
| *EC*0/*EC*1 | 0.02 | 0.01 | 31192 | 3.82 | 0.0001 | 0.07 | 0.04 | 0.11 |

*Note:* The sigma used for effect sizes was 0.28.

**Table S2**

*Contrast test results for ANOVA 2.*

|  | Effect size | | | | | Cohen’s *d* | | |
| --- | --- | --- | --- | --- | --- | --- | --- | --- |
| Contrast | Estimate | SE | df | *t* ratio | *p* value | Cohen's *d* | Lower CL | Upper CL |
| *N*50/*N*200 | -0.17 | 0 | 31192 | -42.71 | <.0001 | -0.69 | -0.73 | -0.66 |
| *N*50/*N*350 | -0.26 | 0 | 31192 | -62.37 | <.0001 | -1.07 | -1.10 | -1.03 |
| *N*50/*N*500 | -0.29 | 0 | 31192 | -65.45 | <.0001 | -1.21 | -1.25 | -1.17 |
| *N*200/*N*350 | -0.09 | 0 | 31192 | -22.84 | <.0001 | -0.37 | -0.41 | -0.34 |
| *N*200/*N*500 | -0.12 | 0 | 31192 | -29.74 | <.0001 | -0.51 | -0.55 | -0.48 |
| *N*350/*N*500 | -0.03 | 0 | 31192 | -8.59 | <.0001 | -0.14 | -0.17 | -0.11 |
| *S*10/*S*25 | 0.11 | 0 | 31192 | 28.38 | <.0001 | 0.46 | 0.42 | 0.49 |
| *S*10/*S*40 | 0.16 | 0 | 31192 | 39.77 | <.0001 | 0.67 | 0.64 | 0.71 |
| *S*25/*S*40 | 0.05 | 0 | 31192 | 15.62 | <.0001 | 0.22 | 0.19 | 0.25 |
| *EG*0/*EG*1 | -0.06 | 0 | 31192 | -15.10 | <.0001 | -0.25 | -0.28 | -0.22 |
| *EC*0/*EC*1 | 0.19 | 0 | 31192 | 42.27 | <.0001 | 0.79 | 0.76 | 0.83 |

*Note.* The sigma used for effect sizes was 0.24.

**Table S3**

*Contrast test results for ANOVA 3.*

|  | Effect size | | | | | Cohen’s *d* | | |
| --- | --- | --- | --- | --- | --- | --- | --- | --- |
| Contrast | Estimate | SE | df | *t* ratio | *p* value | Cohen's *d* | Lower CL | Upper CL |
| Maximax/Maximin | 0.05 | 0 | 31195 | 9.81 | <.0001 | 0.18 | 0.14 | 0.21 |
| Maximax/Minimax | -0.15 | 0 | 31195 | -30.98 | <.0001 | -0.56 | -0.59 | -0.52 |
| Maximax/Minimin | 0.23 | 0 | 31195 | 45.93 | <.0001 | 0.82 | 0.79 | 0.86 |
| Maximax/Random | 0.26 | 0 | 31195 | 53.59 | <.0001 | 0.96 | 0.92 | 0.99 |
| Maximin/Minimax | -0.20 | 0 | 31195 | -40.79 | <.0001 | -0.73 | -0.77 | -0.69 |
| Maximin/Minimin | 0.18 | 0 | 31195 | 36.12 | <.0001 | 0.65 | 0.61 | 0.68 |
| Maximin/Random | 0.22 | 0 | 31195 | 43.77 | <.0001 | 0.78 | 0.75 | 0.82 |
| Minimax/Minimin | 0.38 | 0 | 31195 | 76.91 | <.0001 | 1.38 | 1.34 | 1.41 |
| Minimax/Random | 0.42 | 0 | 31195 | 84.56 | <.0001 | 1.51 | 1.48 | 1.55 |
| Minimin/Random | 0.04 | 0 | 31195 | 7.65 | <.0001 | 0.14 | 0.10 | 0.17 |

*Note.* Tukey’s method was used when comparing a family of five estimates. The sigma used for effect sizes was 0.27.

**Table S4**

*Contrast test results for ANOVA 4.*

|  | Effect size | | | | | Cohen’s *d* | | |
| --- | --- | --- | --- | --- | --- | --- | --- | --- |
| Contrast | Estimate | SE | df | *t* ratio | *p* value | Cohen's *d* | Lower CL | Upper CL |
| Maximax/Maximin | 0.03 | 0 | 31195 | 5.94 | <.0001 | 0.11 | 0.07 | 0.14 |
| Maximax/Minimax | -0.29 | 0 | 31195 | -58.70 | <.0001 | -1.05 | -1.09 | -1.01 |
| Maximax/Minimin | 0.04 | 0 | 31195 | 8.03 | <.0001 | 0.14 | 0.11 | 0.18 |
| Maximax/Random | 0.15 | 0 | 31195 | 30.19 | <.0001 | 0.54 | 0.51 | 0.58 |
| Maximin/Minimax | -0.32 | 0 | 31195 | -64.64 | <.0001 | -1.16 | -1.19 | -1.12 |
| Maximin/Minimin | 0.01 | 0 | 31195 | 2.09 | 0.22 | 0.04 | 0.00 | 0.07 |
| Maximin/Random | 0.12 | 0 | 31195 | 24.25 | <.0001 | 0.43 | 0.40 | 0.47 |
| Minimax/Minimin | 0.33 | 0 | 31195 | 66.73 | <.0001 | 1.19 | 1.16 | 1.23 |
| Minimax/Random | 0.44 | 0 | 31195 | 88.89 | <.0001 | 1.59 | 1.55 | 1.63 |
| Minimin/Random | 0.11 | 0 | 31195 | 22.16 | <.0001 | 0.40 | 0.36 | 0.43 |

*Note.* Tukey’s method was used when comparing a family of five estimates. The sigma used for effect sizes was 0.27.

**Appendix C: Experiment 2 Statistical Outcomes**

In this section, we describe the statistical outcomes for Experiment 2 that were omitted from the main text for brevity. Experiment 2 replicated the findings from Experiment 1 with a reduced set of conditions, because not all combinations of conditions gave rise to noteworthy differences. We briefly describe the four fitted ANOVA models, which are identical in structure to those specified in Experiment 1. The first two tables (Tables S5 and S6) describe the post hoc tests used to evaluate whether different research-condition combinations impacted each of the success metrics, while the latter two tables (Tables S7 and S8) compare differences in algorithm performance for these same metrics.

Our ANOVA models, along with their corresponding tables, can be summarized as follows. First, both ANOVA 5 and ANOVA 6 revealed significant effects for sample size, number of slots, *FlexibleGroups*, and *ExcessCapacity*, with the following results across each:

- Sample size: *F*(1, 51995) = 16946.8, *p* < 0.001, *η*² = 0.25; larger sample sizes significantly improved allocation success, highlighting the model’s reliance on adequate participant pools to optimize allocations.
- Slot numbers: *F*(1, 51995) = 2060.3, *p* < 0.001, *η*² = 0.04; more slots led to decreased allocation success, suggesting the model’s sensitivity to resource complexity and its limitations in balancing multiple options effectively.
- *FlexibleGroups*: *F*(1, 51995) = 1100.6, *p* < 0.001, *η*² = 0.02; alignment between group sizes and slot availability significantly enhanced allocation success, emphasizing the model’s strength in scenarios with well-matched conditions.
- *ExactCapacity*: *F*(1, 51995) = 1297.6, *p* < 0.001, *η*² = 0.02; greater capacity improved allocation outcomes, highlighting the model’s effectiveness in environments with flexibility and surplus resources.

Second, ANOVA 7 and ANOVA 8 demonstrated significant differences in algorithm performance, specifically and respectively: *F*(4, 51995) = 2151.5, *p* < 0.001, *η*² = 0.14, and *F*(4, 51995) = 896.43, *p* < 0.001, *η*² = 0.06. These differences reflect the strengths and limitations of each algorithm in managing allocations.

**Table S5**

*Contrast test results for ANOVA 5.*

|  | Effect size | | | | | Cohen’s *d* | | |
| --- | --- | --- | --- | --- | --- | --- | --- | --- |
| Contrast | Estimate | SE | df | *t* ratio | *p* value | Cohen's *d* | Lower CL | Upper CL |
| *N*50/*N*500 | -0.41 | 0.00313 | 51995 | -130.18 | <.0001 | -1.60 | -1.62 | -1.57 |
| *S*10/*S*40 | 0.13 | 0.00292 | 51995 | 45.39 | <.0001 | 0.52 | 0.50 | 0.54 |
| *EG*0/*EG*1 | 0.11 | 0.00318 | 51995 | 33.18 | <.0001 | 0.41 | 0.39 | 0.44 |
| *EC*0/*EC*1 | 0.15 | 0.00407 | 51995 | 36.02 | <.0001 | 0.58 | 0.54 | 0.61 |

*Note.* The sigma used for effect sizes was 0.26.

**Table S6**

*Contrast test results for ANOVA 6.*

|  | Effect size | | | | | Cohen’s *d* | | |
| --- | --- | --- | --- | --- | --- | --- | --- | --- |
| Contrast | Estimate | SE | df | *t* ratio | *p* value | Cohen's *d* | Lower CL | Upper CL |
| *N*50/*N*500 | -0.29 | 0.00207 | 51995 | -140.31 | <.0001 | -1.72 | -1.75 | -1.70 |
| *S*10/*S*40 | 0.08 | 0.00193 | 51995 | 41.45 | <.0001 | 0.48 | 0.45 | 0.50 |
| *EG*0/*EG*1 | -0.01 | 0.00210 | 51995 | -6.70 | <.0001 | -0.08 | -0.11 | -0.06 |
| *EC*0/*EC*1 | 0.44 | 0.00269 | 51995 | 165.38 | <.0001 | 2.64 | 2.60 | 2.68 |

*Note.* The sigma used for effect sizes was 0.17.

**Table S7**

*Contrast test results for ANOVA 7.*

|  | Effect size | | | | | Cohen’s *d* | | |
| --- | --- | --- | --- | --- | --- | --- | --- | --- |
| Contrast | Estimate | SE | df | *t* ratio | *p* value | Cohen's *d* | Lower CL | Upper CL |
| Maximax/Maximin | 0.11 | 0.00475 | 51995 | 23.20 | <.0001 | 0.32 | 0.29 | 0.35 |
| Maximax/Minimax | -0.07 | 0.00475 | 51995 | -14.51 | <.0001 | -0.20 | -0.23 | -0.17 |
| Maximax/Minimin | 0.23 | 0.00475 | 51995 | 48.91 | <.0001 | 0.68 | 0.65 | 0.71 |
| Maximax/Random | 0.31 | 0.00475 | 51995 | 64.29 | <.0001 | 0.89 | 0.86 | 0.92 |
| Maximin/Minimax | -0.18 | 0.00475 | 51995 | -37.71 | <.0001 | -0.52 | -0.55 | -0.50 |
| Maximin/Minimin | 0.12 | 0.00475 | 51995 | 25.71 | <.0001 | 0.36 | 0.33 | 0.38 |
| Maximin/Random | 0.20 | 0.00475 | 51995 | 41.09 | <.0001 | 0.57 | 0.54 | 0.60 |
| Minimax/Minimin | 0.30 | 0.00475 | 51995 | 63.42 | <.0001 | 0.88 | 0.85 | 0.91 |
| Minimax/Random | 0.37 | 0.00475 | 51995 | 78.81 | <.0001 | 1.09 | 1.06 | 1.12 |
| Minimin/Random | 0.07 | 0.00475 | 51995 | 15.38 | <.0001 | 0.21 | 0.19 | 0.24 |

*Note.* Tukey’s method was used when comparing a family of five estimates. The sigma used for effect sizes was 0.34.

**Table S8**

*Contrast test results for ANOVA 8.*

|  | Effect size | | | | | Cohen’s *d* | | |
| --- | --- | --- | --- | --- | --- | --- | --- | --- |
| Contrast | Estimate | SE | df | *t* ratio | *p* value | Cohen's *d* | Lower CL | Upper CL |
| Maximax/Maximin | 0.05 | 0.00492 | 51995 | 10.55 | <.0001 | 0.15 | 0.12 | 0.17 |
| Maximax/Minimax | -0.11 | 0.00492 | 51995 | -22.74 | <.0001 | -0.32 | -0.34 | -0.29 |
| Maximax/Minimin | 0.12 | 0.00492 | 51995 | 23.44 | <.0001 | 0.32 | 0.30 | 0.35 |
| Maximax/Random | 0.15 | 0.00492 | 51995 | 31.25 | <.0001 | 0.43 | 0.41 | 0.46 |
| Maximin/Minimax | -0.16 | 0.00492 | 51995 | -33.29 | <.0001 | -0.46 | -0.49 | -0.43 |
| Maximin/Minimin | 0.06 | 0.00492 | 51995 | 12.89 | <.0001 | 0.18 | 0.15 | 0.21 |
| Maximin/Random | 0.10 | 0.00492 | 51995 | 20.70 | <.0001 | 0.29 | 0.26 | 0.31 |
| Minimax/Minimin | 0.23 | 0.00492 | 51995 | 46.18 | <.0001 | 0.64 | 0.61 | 0.67 |
| Minimax/Random | 0.27 | 0.00492 | 51995 | 53.98 | <.0001 | 0.75 | 0.72 | 0.78 |
| Minimin/Random | 0.04 | 0.00492 | 51995 | 7.81 | <.0001 | 0.11 | 0.08 | 0.14 |

*Note.* Tukey’s method was used when comparing a family of five estimates. The sigma used for effect sizes was 0.36.

**Appendix D: Further Computational Considerations**

Here, we describe the mathematical and computational limitations and implications of the work presented, which could not be accommodated in the main text. For example, we describe how the commitment to realism in the research design may have hindered a full dissemination of effects, how differing sampling procedures could have impacted our results, and shortcomings in the algorithm designs. We also discuss how all of these aspects could be improved and suggest what more sophisticated algorithms might look like in the future.

One major limitation of the research presented here is the omission of single-slot appointments as part of the simulation grids. It is possible that the heuristics presented here would also provide reasonably high-quality allocation solutions to appointments for individuals as well as groups. This could have allowed for an interesting simulation and comparison of self-organizing participant behavior (where participants select one appointment that suits them) with allocation strategies (where an algorithm makes the decision with the participant’s preferences in mind). However, given the trends observed in Experiment 1, it seems likely that the algorithms would not have fared as well with individual appointments owing to them being a maximally extreme case of many slots being present with no flexibility for slot sizes. This caveat brings three further limitations to mind, namely (1) the use of very simple strategies that make no inferences about the subjective preferences and motivations of individuals, (2) the lack of post hoc optimization strategies, and (3) the lack of purpose-built algorithms for specific tasks. Regarding the first of these, it seems plausible to suggest that the algorithms presented here still offer considerable opportunity for further optimization. For example, the heuristics of this study are indifferent as to who is allocated to a slot once potential participants have been identified. It is very possible that a single metric that quantifies how difficult it is to accommodate an individual's preferences (based on the number of selections and their popularity) would allow the heuristic to make far better allocation decisions.

Furthermore, the strategies could be expanded into metaheuristics that have parameter estimates and exhibit different behaviors depending upon the number of participants that remain to be allocated. One such example could be a metaheuristic that becomes increasingly greedy as the number of people still unallocated decreases, preferring to allocate to existing, partially filled time slots rather than starting to fill ones currently empty. Another avenue to improve these algorithms could be the use of post hoc optimization strategies. A proposed solution could be iterated over by, for example, swapping participants between time slots in order to make room for people as yet unallocated. Alternatively, the computational efficiency of heuristics means that they could easily be run hundreds or thousands of times with random number generation, with only the best outcome being put forward to the user as the solution. Because of their relatively low computational burden and comparably high evident ability to find desirable solutions, this use of heuristics would still be preferable to brute-force computation. On a more psychological note, algorithms might also benefit from taking advantage of the structured dynamics of subjective human preferences; for example, by weighting some slots (e.g., outside conventional work hours) more highly for those participants showing an interest in them.

A different set of limitations stems directly from the simulation procedure employed here. The parameter grid of the simulation was meant to reflect a variety of realistic real-world research scenarios. However, this commitment to realism inadvertently limits how generalizable certain findings are. For example, the effects that were observed in situations that featured excess time-slot capacity may be an artifact of the circumstance that all simulations with small sample size (*N* = 50) also had excess time-slot capacity. Thus, it is not deducible from the set of simulations featured here whether the presence of excess capacity intrinsically affects algorithm performance or whether this effect is simply induced by small sample sizes. A second simulation-procedure-related flaw of this study is the difference in data generation between Experiments 1 and 2. Experiment 1 utilized randomly generated datasets but Experiment 2 utilized nonparametrically bootstrapped samples from a participant pool. This difference restricts the opportunity to distinguish effects between simulated and real-world data because bootstrapped samples from a comparatively small pool are likely to have greater similarity to one another than samples that were randomly generated independently. Although it was possible to identify likely causes of misalignment despite this limitation, it would have been desirable to generate a set of individuals from which to bootstrap samples in Experiment 1 too. Moreover, bootstrapping samples for Experiment 1 would certainly have reduced the computational burden of the simulations. On the other hand, the use of bootstrapping to increase the statistical power of Experiment 2 necessarily meant that the sample used was less variable than a natural sample of equal size. We attempted to address this concern by recruiting as large a pool of real participants from which to source the bootstrapped samples as possible.

A final set of limitations relates to the nature of the heuristic algorithms developed as part of this research. All four algorithms essentially filter the set of participants according to a popularity condition and then allocate a specific number of remaining eligible individuals indiscriminately. As such, the heuristics have relatively minor differences from one another, meaning that the research may lack model diversity. As such, findings related to the allocation behavior of the heuristics overall may not be generalizable to strategies that differ from those demonstrated here: it is entirely possible that inherently different strategies would exhibit diverging patterns. Finally, it should be noted that the heuristics presented as part of this research were constructed as general rules, rather than purpose-built ones. To elucidate, they were not developed to specialize in particular tasks or specific situations; it is entirely possible that sizeable performance improvements could be observed when developing strategies specific to, for example, the Max-set case.

There are several avenues for future research based on the findings of this study. As mentioned in the Limitations section of the main article, there is the opportunity to develop more diverse sets of algorithms that utilize novel metrics. One plausible new feature could be the relative inflexibility of the participant. It stands to reason that a person who is maximally inflexible (i.e., has only selected a single time slot) should be prioritized because otherwise they may remain unallocated, while those with greater numbers of indicated choices are easier to accommodate later on in the allocation procedure. Finally, given the findings of the exploratory analysis of Experiment 2, there is the possibility of improving the simulation technique to better account for people’s tendency to reduce their effort investment when forced to persist with an arduous task and their relatively lower tendency to be correlated in their choices to other people . Simulations could also be expanded to take into consideration other sample constraints, such as individual differences in the participants that make up a group, or some predictive model of participant dropout. Other promising avenues of future research could use more sophisticated decision rules, include additional local optimization procedures, and tailor algorithms to specific use-cases.
